# The revision of the 2014 European tobacco products directive: an analysis of the tobacco industry's attempts to ‘break the health silo’

**DOI:** 10.1136/tobaccocontrol-2014-051919

**Published:** 2015-02-24

**Authors:** Silvy Peeters, Hélia Costa, David Stuckler, Martin McKee, Anna B Gilmore

**Affiliations:** 1 Department for Health, University of Bath, and member of the UK Centre for Tobacco and Alcohol Studies (UKCTAS), UK; 2 Department of Sociology, University of Oxford, Oxford, UK; 3 Department of Public Health and Policy, London School of Hygiene & Tropical Medicine, London, UK

**Keywords:** Public policy, Tobacco industry, Tobacco industry documents

## Abstract

**Background:**

The 2014 European Union (EU) Tobacco Products Directive (TPD) was negotiated in a changed policy context, following adoption of the EU's ‘Smart Regulation’ agenda, which transnational tobacco companies (TTCs) anticipated would increase their influence on health policy, and the WHO Framework Convention on Tobacco Control (FCTC), which sought to reduce it. This study aims to explore the scale and nature of the TTCs' lobby against the EU TPD and evaluate how these developments have affected their ability to exert influence.

**Methods:**

Analysis of 581 documents obtained through freedom of information requests, 28 leaked Philip Morris International (PMI) documents, 17 TTC documents from the Legacy Library, web content via Google alerts and searches of the EU institutions' websites, plus four stakeholder interviews.

**Results:**

The lobby was massive. PMI alone employed over 160 lobbyists. Strategies mainly used third parties. Efforts to 'Push' (amend) or 'Delay' the proposal and block 'extreme policy options' were partially successful, with plain packaging and point of sales display ban removed during the 3-year delay in the Commission. The Smart Regulation mechanism contributed to changes and delays, facilitating meetings between TTC representatives (including ex-Commission employees) and senior Commission staff. Contrary to Article 5.3, these meetings were not disclosed.

**Conclusions:**

During the legislative process, Article 5.3 was not consistently applied by non-health Directorates of the European Commission, while the tools of the Smart Regulation appear to have facilitated TTC access to, and influence on, the 2014 TPD. The use of third parties undermines Article 5.3.

## Introduction

Tobacco is Europe's largest preventable cause of death, claiming nearly 700 000 lives in the European Union (EU) annually.^1^ Although the EU's public health legislative powers are limited,^2^ the launch of the 1985 ‘Europe Against Cancer’ programme^3^ prompted a range of tobacco control measures,^4^ including the 2001 Tobacco Products Directive (TPD) (2001/37/EC) which regulates the manufacture, sale and presentation of tobacco products. In 2009, the European Commission (‘the Commission’) began revising this Directive in light of new market and scientific developments and the WHO's Framework Convention on Tobacco Control (FCTC).^5^ The process took over 5 years, with the new Directive finally adopted in April 2014. The Directive, which includes an increase in the size of graphic health warnings, a ban on characterising flavours, restrictions on the size and shape of cigarette packs, and the regulation of Electronic Nicotine Delivery Systems (ENDS) (table 1), must be transposed into national law by 2016.^6^

**Table 1:** Textual changes to the 2014 TPD

Key provisions	Commission proposal 19/12/2012	Council common approach 21/06/2013	Parliamentary health committee approved text 10/07/2013	Parliamentary plenary approved text 8/10/2013	Trilogue agreement (between commission, council and parliament) 18/12/2013
Size and position of health warnings	75% front, back and top MS discretion	65% front, back and top MS discretion	75% front, back and top MS discretion	65% front, back and top MS discretion	65% front, back and top MS discretion
Ban on ‘characterising flavours’	Yes	Yes	Yes	Yes, menthol 5 years derogation	Yes, menthol 4 years derogation
Slim cigarette ban	Yes	No	Yes	No	No
Ban on 10 cigarette pack	Yes	Yes	Yes	Yes	Yes
Cross border distance sales	Notification, mandatory age verification	Prohibit or notification MS discretion	Prohibit	Prohibit	Notification MS discretion
Traceability and security features	Track and trace to extend to the whole supply chain	Track and trace to extend to the whole supply chain	Track and trace to extend to the whole supply chain. No tobacco industry solutions	Track and trace to extend to the whole supply chain. No tobacco industry solutions	Track and trace system for the legal supply chain
Snus sales ban	Maintained	Maintained	Maintained	Maintained	Maintained
ENDS regulation	Medicines licence, depending on nicotine concentration	Medicines licence, depending on nicotine concentration	Medicines licence all	No, only if they make health claims	No, only if they make health claims

MS, Member States.

While these changes represent significant public health advances, the final Directive is weaker than initial drafts^7^ (table 1). The review process involved controversy, notably the forced resignation of Health Commissioner John Dalli and claims of tobacco industry interference,^8–12^ with the TPD described as ‘the most lobbied dossier in the history of the EU institutions’.^13^ Although previous research reveals transnational tobacco companies’ (TTCs) efforts to derail earlier EU tobacco regulation,^2^ ^14–16^ the policy context has since changed in ways that may mitigate or exacerbate TTCs’ ability to influence EU legislation. On the one hand, FCTC Article 5.3 entered into force in 2005, requiring that ‘in setting and implementing their public health policies with respect to tobacco control, parties shall act to protect these policies from commercial and other vested interests of the tobacco industry’.^17^ Conversely, regulatory reforms known in the EU as Better or Smart Regulation, and shown to facilitate tobacco industry influence,^18^ ^19^ were implemented in the mid-2000s.^20^ Smart Regulation seeks to reduce regulatory burdens and enhance business competitiveness via impact assessment (IA), which attempt to estimate the costs and benefits of policies in monetary terms, and stakeholder consultation in which those affected by the policy are formally consulted early in the policy process. Worryingly, British American Tobacco (BAT), working with a large number of other corporations whose products are potentially damaging to health, was instrumental in promoting Smart Regulation, anticipating it would make it harder to enact public health legislation.^21^ In line with BAT's predictions, growing evidence suggests that Smart Regulation can^19^ ^21^ ^22^ and has^23^ ^24^ favoured corporate interests and might undermine efforts to implement public health policies.^19^ ^21^ ^22^

We previously demonstrated, using quantitative content analysis, that successive drafts of the TPD shifted towards the tobacco industry's preferred position.^7^ We explore how the tobacco industry engineered some of these policy changes. We examine the nature and scale of TTCs’ efforts to influence the TPD revision, identifying key entry points used to access and shape the policy process. We also examine whether Smart Regulation enabled corporate influence on the TPD, as those promoting it intended,^21^ and whether the application of Article 5.3 is adequate to prevent TTC influence on EU tobacco control policymaking.

## Methods

We analysed a wide variety of materials. First, we obtained 2007–2014 reports, meeting minutes, and press releases from the Commission (http://ec.europa.eu/health/tobacco/policy/index_en.htm), Council of Ministers (http://www.consilium.europa.eu/homepage) and European Parliament (http://www.europarl.europa.eu/news/en/news-room/) websites. Second, relevant web content (including press coverage, media releases and, blogs) was identified prospectively through Google alerts established in 2011 on BAT, ‘Philip Morris International’ (PMI), ‘Japan Tobacco International’, ‘Imperial Tobacco’, ‘Swedish Match’, and ‘Tobacco Products Directive’. Third, internal TTC documents were taken from two sources: 28 PMI documents detailing its strategy to influence the TPD, dated 2011–2013 and leaked to health groups in 2013 (‘PMI's documents’), and 17 (of 323 retrieved) documents obtained from the Legacy Tobacco Documents Library (http://legacy.library.ucsf.edu/) (‘Legacy documents’) using search terms ‘tobacco products directive’, ‘TPD’, ‘tobacco directive’ and ‘impact assessment’, and document dates 2007–2013.

Finally, to triangulate the industry documents, identify whether actions detailed were carried out and further examine the TTCs’ political activity, we analysed documents, all dated 2010–2013, released under EU Freedom of Information (FOI) legislation (n=581) or given to the authors (n=2) (‘FOI documents’) and undertook four stakeholder interviews. The majority of the FOI documents were released directly to the authors (n=425) or their contacts (n=109) and are previously unpublished, while others (n=47) were available online at http://www.asktheeu.org (see online supplementary appendix 1).

Documents were analysed using a deductive hermeneutic approach,^25–27^ that is, understanding documents’ content in a wider policy context, while identifying conceptual themes and subthemes (eg, corporate political strategies and tactics) which were repeatedly tested as data collection progressed. Events and meetings, identified from the documents and occurring from 2007 to 2014, were recorded in a timeline to map key developments, identify stakeholders and points of access to EU institutions, and time the Directive's progress through the legislative process. We compared the time the 2014 TPD revision took in each legislative stage, that is, in the Commission, then Parliament and Council, with the original 2001 TPD.

Semistructured interviews were undertaken with staff of the most active Brussels-based tobacco control NGO, the Smoke Free Partnership (SFP) and three Members of European Parliament (MEPs) (Twelve MEPs identified in the European Parliament's TPD ‘procedural file’ as key players^28^ were invited for interview, but only three accepted). Staff of DG-SANCO, the Commission's department responsible for tobacco control, were also approached but declined. Interviews were professionally transcribed and coded using a thematic approach based on the literature while also allowing for new themes to emerge.^29^

## Results

### Tobacco industry strategy

PMI's approach to the 2014 Directive was to either ‘Push’ (ie, amend) or ‘Delay’ the proposal, or block any ‘extreme policy options’ which it identified as standardised packaging, a point of sales display ban and an ingredients ban.^30^ ^31^ Its strategy detailed actions to be taken at each stage of the legislative process. In the Commission, PMI aimed to “Block DG SANCO's extreme policy options”,^30^ in Parliament to “Break ENVIs (Health Committee) full control on the dossier”,^30^ and in the Council to “Create [a] blocking minority to any extreme measures”.^30^

#### David versus Goliath

PMI alone employed more than 160 lobbyists and spent €1.25 million on lobbying to subvert the TPD.^32^ At least seven tobacco industry lobbyists were former EU politicians or civil servants.^33^ ^34^ By contrast, Brussels-based health advocates had five fulltime equivalent positions working on the TPD, with a slight increase when the proposal was published in December 2012 (personal correspondence, SFP 12 May 2014). Comparing the health to the tobacco lobby, one MEP likened it to a biblical battle: ‘if you see who is fighting on the left hand side and who is fighting on the right hand side…then you get a shock. It is David and Goliath. It's unbelievable’ (interview MEP, January 2014).

#### Third party mobilisation

PMI's first ‘principle’ for achieving its objective was ‘indirect engagement over direct engagement’,^31^ describing third party involvement as ‘key to success’.^35^ PMI sought to use a ‘3rd party coalition’ to garner political support from non-health Commissioners using four frames or ‘platforms’: intellectual property, ingredients, retailers and smokeless tobacco.^30^ ^36^ PMI named 15 associations and 2 companies as coalition members, including the European Tobacco Growers Association (Unitab), European Federation of Food, Agriculture and Tourism Trade Unions (EFFAT), and the European Federation of Tobacco Processors (FETRATAB) leading on the ingredients’ platform, and the European Association of Tobacco Retailers (CEDT) on the retailers' platform.^36^

FOI documents and Parliamentary and Commission meeting minutes and reports confirm that 12 of PMI's third party coalition partners (11 associations and 1 private company), were actively involved in lobbying the Commission and Parliament, and mobilising opposition.^37–45^ For example, CEDT established a European retailers’ TPD Working Party which mobilised member state retail organisations.^46^ ^47^ In addition, we identified 126 associations and 33 non-TTC companies (17 public relations and law firms) that voiced opposition to the TPD, through industry stakeholder meetings with the Commission,^37^ ^48^ approaching Commission officials and MEPs with their concerns,^49–52^ participating in working groups to develop counterstrategies or policy statements,^46^ ^53^ ^54^ making critical statements in the media,^55–58^ and signing anti-TPD petitions^59^ (see online supplementary appendix 2).

**Figure 1:**
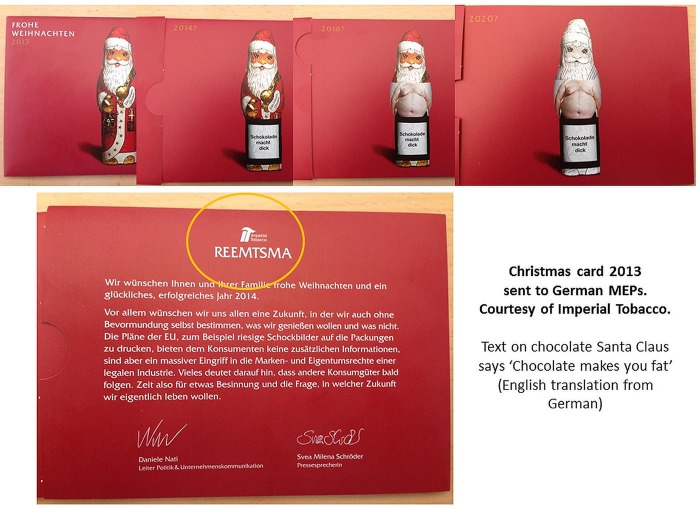
Gifts sent to Members of European Parliament (MEPs) in 2013 to stress the danger of possible ‘spill-over’ effects of Tobacco Products Directive (TPD) measures into the alcohol and food industries.

In ongoing work, we have identified 51 of 137 third party associations as having financial links with the tobacco industry. Their interests and actions are consistent with PMI's strategy.^36^ For example, many (44) were general business associations and FOI documents reveal that PMI and BAT attended meetings organised by Business Europe and the Dutch business association, VNO-NCW, intending to build a Europe-wide business lobbying presence to stress the TPD's ‘spill-over’ effects on other sectors.^53^ ^54^ This spill-over effect (eg, on food and alcohol industries) was emphasised in TTC lobbying of MEPs (figure 1). Importantly, meeting minutes specify that the business groups would not attend a formal tobacco industry stakeholder meeting hosted by DG-SANCO, to create the perception of autonomy from the tobacco sector.^53^

### Delays in the commission policy process

**Figure 2:**
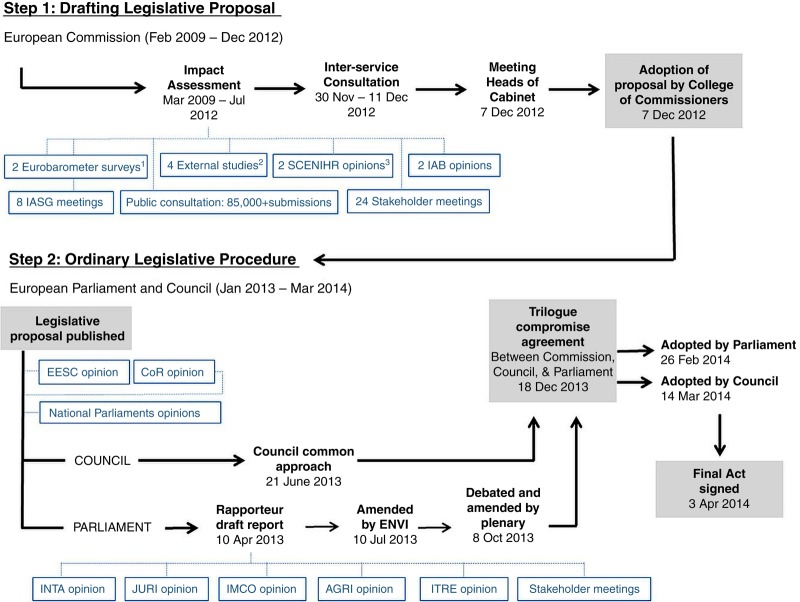
Legislative process undertaken to review the Tobacco Products Directive (TPD). Source: summary of data collected from the websites of the European Commission, Parliament and Council, accessed regularly between May 2012 and April 2014. AGRI, Committee on Agriculture and Rural Development; CoR, Committee of the Regions; EESC, European Economic and Social Committee; ENVI, Committee on the Environment, Public Health and Food Safety; IAB, Impact Assessment Board; IASG, Impact Assessment Steering Group; INTA, Committee on International Trade; IMCO, Committee on Internal Market and Consumer Protection; ITRE, Committee on Industry, Research and Energy; JURI, Committee on Legal Affairs; SCENIHR, Scientific Committee on Emerging and Newly Identified Health Risks. ^1^Special Eurobarometer 332 (May 2010), Special Eurobarometer 385 (May 2012). ^2^GHK Consulting, *A Study on Liability and the Health Costs of Smoking, Final Report* (December 2009), Rand Europe, *Assessing the impacts of Revising the Tobacco Products Directive. Study to support a DG SANCO Impact Assessment, Final Report* (September 2010), RAND Europe, *Availability, accessibility, usage and regulatory environment for novel and emerging tobacco, nicotine or related products* (December 2012), Matrix Insight, *Economic analysis of the EU market of tobacco, nicotine and related products* (September 2013). ^3^Smokeless tobacco (February 2008), Additives in Tobacco Products (November 2010).

The 2014 TPD followed the usual legislative procedure (figure 2). However, a comparison with the 2001 TPD shows that, while each Directive spent an equal amount of time in the codecision stage, the 2014 Directive spent 3 years longer with the Commission.

As the proposal needed to be adopted before the Parliamentary elections in May 2014, this slow progress is significant. We identify several potential reasons for it.

#### The IA stage

In line with the EU's Smart Regulation agenda, and unlike the 2001 Directive which included only a brief IA,^15^ the 2014 TPD revision was subjected to a comprehensive IA. This was not finalised until mid-2012, long beyond the anticipated completion in late 2010.^60^ Two developments likely contributed to this delay. First, after strong industry opposition to a RAND Europe study that provided the baseline for the Commission's IA,^61^ DG-SANCO commissioned two further studies,^62^ ^63^ which had not been anticipated in the official roadmap.^60^ Second, DG-SANCO's public consultation on the Directive attracted over 85 000 submissions, more than any other EU consultation. The Commission attributed this unprecedented response, 57% of which were duplicates, to tobacco industry-led mobilisation campaigns in Italy and Poland,^64^ while PMI's documents also indicate the industry's role by revealing that the majority of the 85 000+ submissions were ‘known’ to PMI.^65^

#### Delays to the Inter-Service Consultation

**Figure 3:**
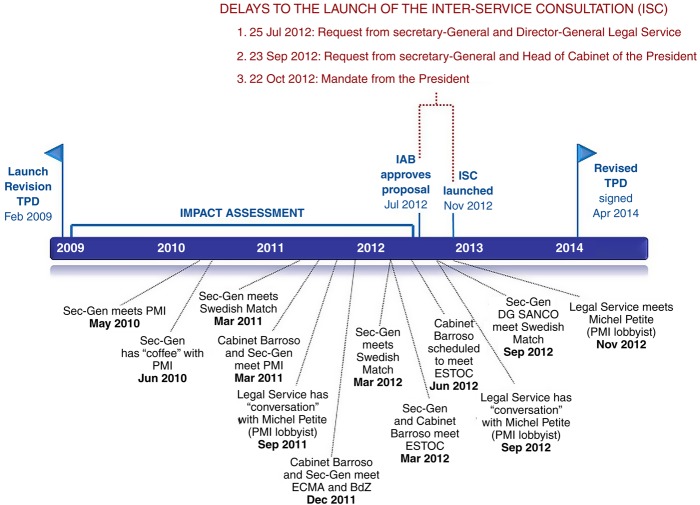
Delays to the Inter-Service Consultation and linked Undisclosed ‘Meetings’* with the Tobacco Industry. Source: Letters and emails released under Freedom of information requests^146–148^ and Parliamentary Inquiry.^75^ BdZ, German Cigar Manufacturers Association; ECMA, European Cigar Manufacturers Association; ESTOC, European Smokeless Tobacco Council; IAB, Impact Assessment Board; ISC, Inter-Service Consultation; PMI, Philip Morris International; Sec-Gen, Secretariat-General; TPD, Tobacco Products Directive. *It is not known whether all meetings occurred in person.

FOI documents identify three specific delays to the Inter-Service Consultation (ISC), the Commission's internal consultation with Directorates-General (DGs) affected by the proposal, linked to events at the highest level of the Commission (figure 3).

First, Secretary-General Catherine Day (the most senior EU civil servant) and Legal Service's Director-General Luis Romero Requena requested that DG-SANCO postpone the ISC launch scheduled for 22 August 2012^68–70^ because there were “A number of substantial issues needing further attention”.^66^ They claimed that, despite the draft IA having been approved by the Commission's Impact Assessment Board (IAB) on 12 July 2012, not all issues raised by the IAB had been addressed.^66^ They also alleged concerns with the proposal's legal basis, an argument identified in a 17 August 2012 PMI document as appealing to the ‘sensitivities’ of the Secretariat-General and the Legal Service (see below).^30^ Emails between DG-SANCO and Day on 7 September suggest that, as a result, DG-SANCO removed plain packaging and the point of sale display ban from the proposal.^71^ Second, the ISC launch was further delayed on 23 September following concerns by Day and the Chief of Barroso's Cabinet, Johannes Laitenberger, that “it would be best not to launch the ISC until after the October European Council—this is a text that might well leak even from ISC and we are keen to avoid too much controversy before [then]”.^72^ Third, on 16 October, days before the rescheduled ISC launch on 22 October, Commissioner John Dalli was forced to resign by President Barroso in an opaque cash-for-access scandal, with Barroso mandating that the ISC should wait until a new Health Commissioner was in place.^73^ ^74^ Ultimately new Commissioner Tonio Borg launched the ISC 2 days after taking office, on 30 November 2012, 3 months later than originally intended.

Despite FCTC Article 5.3, FOI documents show that the Secretariat-General, the Legal Service, and Barroso's Cabinet held at least 12 TPD-related ‘meetings’ with the tobacco industry between 2010 and 2012 (figure 3). Unlike DG-SANCO's practice, in line with Article 5.3, of publishing minutes of stakeholder meetings on the Commission's website, none of these meetings were publicly disclosed. This includes contact from September 2011 to November 2012 between the Legal Service and Michel Petite who, until 2008, was Director-General of the Commission's Legal Service but, when the meetings occurred, was a consultant to PMI in his role at law firm, Clifford Chance. Despite Legal Service being aware of Petite's new role,^75^ Petite was twice able to “set out his views on some legal issues of tobacco legislation” and meet with the Legal Service Director-General.^75^ This is notable because John Dalli claims that Barroso asked him to shelve the TPD in November 2011 because “his [Barroso's] legal services were raising many legal issues”, and that DG-SANCO officials advised him that the Legal Service only started raising concerns following Petite's involvement.^70^

#### Cash-for-access controversy: ‘Dalligate’ or ‘Barrosogate’

A further delay within the Commission occurred following the ‘Dalligate’ controversy,^76^ which some MEPs relabelled ‘Barrosogate’.^77^ For details see TobaccoTactics.org.^78^ Petite, acting as proxy for snus manufacturer Swedish Match (which has a joint venture with PMI^79^), approached Day in March 2012 alleging that Dalli's business associate, Silvio Zammit, tried to solicit €60 million from Swedish Match in return for Dalli lifting the snus sales ban that was included in the 2001 TPD but was being reconsidered in the revision.^80^ After a written complaint by Swedish Match in May 2012, Day referred the matter for investigation to Giovanni Kessler, Director-General of the EU Anti-Fraud Office (OLAF), and shared the complaint with Romero Requena, Barroso and Laitenberger.^75^

On 15 October 2012, OLAF finalised its investigation and forwarded its conclusions to Day.^75^ The following day and, crucially, days before the launch of the Commission's ISC, Barroso forced Dalli to resign, with the Commission's press statement stating that OLAF found that Zammit had approached Swedish Match using his contacts with Dalli and sought to gain financial advantages in exchange for influence over a possible future legislative proposal on snus.^81^ The Commission's press statement also noted there was “no conclusive evidence” of Dalli's direct participation, and “no transaction was concluded between the company and the entrepreneur [Zammit] and no payment was made”.^81^ It also emphasised that Dalli maintained his innocence^69^ ^82^ and in June 2013 the Maltese police stated that there was insufficient evidence to prosecute him.^83^ ^84^ At the time of writing, Zammit's trial is still ongoing.

Many questioned whether Dalli's alleged misconduct justified his punishment, given that Dalli had not benefited personally, and the text of the proposal had not changed as a consequence.^85^ ^86^ The Commission's decision came under further scrutiny when the secret OLAF report^87^ was leaked, presenting only circumstantial evidence against Dalli, retrieved using seriously flawed methods.^8^ ^88^ ^89^ OLAF concluded, inter alia, that Dalli's unofficial contacts with snus lobbyists (two meetings in total, both in Malta and occurring at the request of Swedish Match and the European Smokeless Tobacco Council (ESTOC)) had not been publicly disclosed and were a breach of the Commissioner's Code of Conduct and the FCTC's Article 5.3.^87^ Yet senior staff from the Secretariat-General, Legal Service and Barroso's Cabinet met at least 12 times with the tobacco industry (figure 3), without sanction.

#### Commission delays consistent with PMI's strategy

To block ‘extreme policy options’ at the drafting stage, PMI sought to trigger negative opinions from DGs other than DG-SANCO,^30^ with ‘Barroso's Circle’ (ie, Secretariat-General and Barroso's Cabinet) identified as having unequivocal power to intervene.^30^ TTC and third party political activity was targeted at DGs Enterprise, Trade, Agriculture and Rural Development and Internal Market. Perhaps in an attempt to weaken DG-SANCO's position, or to involve the Secretariat-General, TTCs and third parties criticised DG-SANCO's IA and stakeholder consultation,^90^ arguing that it had failed to account for the proposal's ‘unintended consequences’ (ie, illicit trade).^39^ ^91–93^

PMI developed five messages to undermine the TPD proposal that would appeal to other DGs: lack of evidence, lack of logic, lack of acknowledgement of the public consultation response, failure of the IA to adequately assess impacts on the tobacco market, notably illicit trade, and lack of a legal basis.^30^ All except the ‘lack of logic’ message feature repeatedly in FOI documents.^93–98^ FOI documents also show that the Commission received various industry-commissioned technical reports,^99–102^ three of which are identified in PMI's documents as ‘tools’ for strengthening pro-tobacco arguments.^30^ ^103–105^ Two of these reports stressed and costed what the tobacco industry considered ‘negative’ or ‘unintended’ socioeconomic consequences.^103^ ^104^ FOI documents also show that Commissioners and senior officials were invited to tobacco industry events,^47^ ^98^ ^106–109^ including a BAT stakeholder event on harm reduction and illicit trade,^33^ BAT's annual lunch with policy elites,^110^ ^111^ and PMI's launch of KMPG's (heavily criticised^112^) study on illicit trade.^113^

### Progress through the parliament and council in codecision

#### ‘Break ENVI's full control on the dossier’

In January 2013, after 4 years in the Commission, the proposed legislation moved to the Parliament and Council (figure 2). To increase the prominence of market versus public health arguments, PMI encouraged the appointment of the Internal Market Committee (IMCO) as co-lead parliamentary committee, alongside the Health Committee (ENVI) which would normally preside over this file. Interview data, however, suggests that Dalli's departure had resulted in rare, all-party support to move the TPD forward, and that to assign IMCO co-chair status would have led to another scandal (interviews, MEP and health advocate, January 2014). As one MEP recalled, “we guessed that the next tactic would be that they shouldn't give [the proposal] to the environment committee [ENVI]…, but it was really a nonstarter…it would have been another scandal…they shot themselves in the foot with that” (interview MEP, January 2014).

PMI hoped to generate opposition from the five appointed Committees for Opinion (International Trade, Internal Market, Legal Services, Agriculture and Rural Development and Industry Research and Energy).^30^ ^31^ To this end, PMI's documents reveal a lobbying offensive targeting MEPs as early as 2010, when the proposal was still being drafted.^114^ PMI meticulously assessed each MEP's position on TPD policy options and sensitivity to pro-tobacco arguments,^115–117^ identifying ‘heavy weights’ within each political group, particularly the largest centre-right European People's Party (EPP),^118^ and the Committees for Opinion.^119^ PMI's national offices approached MEPs in their constituencies,^114^ where MEPs are more ‘off-guard’ without staff reminding them of protocol (interview MEP, January 2014). By August 2012, when the Commission had finalised the proposal's IA, PMI lobbyists had already met with one-third of MEPs (257 of 754).^30^

PMI's documents note that the Dalli controversy negatively impacted their ability to access policymakers.^31^ FOI documents and interview data confirm that, at least temporarily, it changed the political landscape against the tobacco industry (interviews MEPs and NGO, January 2014). For example, previously amenable DGs^120–124^ became less inclined to engage with the tobacco industry.^125–128^ In Parliament, the EPP, on which PMI heavily relied for support,^118^ decided not to nominate a candidate for TPD rapporteur (the MEP who reports on the proposal and oversees its progress). Instead, in January 2013, Linda McAvan from the Social-Democrats was appointed rapporteur, a choice PMI described as ‘hostile’.^31^ Interview data suggest that lobbying intensified thereafter, with one MEP describing the tobacco lobby as ‘unbelievably powerful’ (interview MEP, January 2014). Whereas PMI's documents reveal that third parties were ‘activated’ to approach health-friendly MEPs, often hiding their tobacco industry links,^35^ interview data suggest that former MEPs were purposively recruited to approach MEPs on the basis of being ‘an old friend’ (interview MEP, January 2014). Tobacco-friendly MEPs also attempted to isolate influential MEPs within their own parties who failed to support industry positions (interview MEP, January 2014).

Various amendments tabled by MEPs appeared to have originated from the tobacco industry,^129^ ^130^ including amendments on ‘delegated acts’ outlined in PMI's documents.^31^ One MEP commented that ‘…amendments that came on most of the articles were clearly not written by the MEPs, and they weren't things they would normally have been aware of’ (interview MEP, January 2014). One MEP observed that it was TTCs’ innovative packaging, including lipstick-style packs targeting young women, which swayed Parliamentary opinion, and led to McAvan being given the mandate to move the TPD forward.

#### ‘National level is key’: working through National Parliaments and the Council

PMI believed the ‘National level is key’.^31^ Thus it tried to influence the Commission's initial proposal via national Health Ministers and their officials on the Commission's TPD Regulatory Committee. For example, PMI Netherlands, which had cultivated a relationship with the Dutch Department of Health,^131–134^ attempted to get the Department to delay the proposal in the Commission, arguing that DG-SANCO's consultation on the RAND report was inadequate and inconsistent with the Commission's IA Guidelines.^132^

PMI also sought to mobilise national parliaments to cause delay through the ‘yellow card’ system, triggered when a sufficient number of national parliaments issue a ‘reasoned opinion’ that the proposal does not comply with the EU's subsidiarity principle.^135^ This failed when only seven reasoned opinions were submitted.^136^ PMI also sought to mobilise a blocking minority in the Council through ‘third party mobilisation’, attempting to frame the debate around employment and small to medium enterprise issues.^30^ However, the Council reached a consensus on 21 June 2013, with only Poland, Bulgaria, Romania and the Czech Republic opposing it.^137^

## Discussion

Our findings demonstrate that the tobacco industry considered the revised TPD a serious threat and mounted a massive lobbying campaign against it. PMI alone employed more than 160 lobbyists and met individually with a third of MEPs before the proposal reached Parliament. Overall the campaign attempted to shift the debate away from health towards alleged negative economic impacts of the proposal and to isolate or weaken those with an interest in health—DG-SANCO and the Health Commissioner within the Commission, and members of the Parliament's ENVI committee. Former EU officials now working or consulting for the tobacco industry played key roles. Lobbying was directed at all three EU institutions, with TTC access and influence in the European Commission secured via its highest echelons, the Secretariat-General, the Legal Service and Barroso's Cabinet. Intervention by the Secretariat-General led both to the removal of the two provisions that industry was most concerned about—plain packaging and a point of sales display ban—and to repeated delays. We also show that these interventions followed repeated, undisclosed contact between senior Commission officials and the tobacco industry in contravention of Article 5.3. As such, PMI's strategy to ‘delay’ or ‘push’ (ie, amend) the proposal appears to have been successful.

The evidence presented cannot provide an exhaustive summary of all lobbying activity aimed at shaping the TPD. Although we benefited from the availability of PMI's documents, we did not have access to similar data sets from other TTCs. Nonetheless, it is clear from FOI documents and interviews that other TTCs were similarly politically active, and at times collaborated. Data were also biased towards TTCs’ political activity in the Commission (through FOI documents), and to a lesser degree Parliament (through interviews), while less is known about TTCs’ political activity in the Council stage and at national level. The small interview sample reflects the reluctance of EU officials to discuss the TPD while it was still being legislated. Further, the study does not examine the influence of public health groups although it is clear that some, particularly the SFP, played a key role in securing the TPD's success.

Our study has several implications for EU policy. First, the EU's Smart Regulation agenda, specifically its requirements for stakeholder consultation and IA, in which the impacts of policies must be assessed and costed and ‘burdens of legislation’ minimised for ‘economic operators’,^19^ allowed the industry to frame arguments, engage Commission staff, and delay the Directive's progress. These findings reflect BAT's aims in promoting Smart Regulation tools in the 1990s.^19^ ^21^ Specifically, the requirement that affected stakeholders be consulted early in the legislative process enabled TTCs to input at the outset and overwhelm the process by mobilising the largest ever response to an EU consultation. The Commission's intention to democratise policymaking through stakeholder consultation^138^ clearly fails to account for the ability of powerful corporate actors to dominate this process. The requirement for a comprehensive IA led to significant delays so that the TPD proposal took 3 years longer in the Commission than the original 2001 Directive.

Second, an important difference in TTCs’ activities in the current versus the 2001 directive^15^ was their extensive use of third-party actors. We identified 137 associations and 34 non-TTC companies that voiced support for policy outcomes favoured by the tobacco industry; 12 were identified by PMI as part of its ‘3rd party coalition’. This increased emphasis on third parties likely reflects an unintended consequence of the adoption of the FCTC's Article 5.3. While DG-SANCO clearly complies with 5.3,^139^ ^140^ other parts of the Commission and some MEPs do not. The fact that senior Commission staff held undisclosed meetings with the tobacco industry, yet cite Article 5.3 as a key reason for Dalli's dismissal shows a misinterpretation and mis-implementation of the Article.

Despite the tobacco industry's success in delaying and amending the 2014 TPD, it was still enacted in April 2014 and significantly advances EU tobacco control. Although plain packaging was removed, pictorial warning labels covering 65% of the pack were implemented and represent an increase of 25–30% from current coverage. Interview data and press coverage^9^ ^12^ ^76^ ^141^ suggest that the industry's aggressive lobbying and its initial receptive response within parts of the Commission, culminating in the forced resignation of Commissioner Dalli, ultimately backfired. Serious questions began to be raised by NGOs about the transparency of EU policymaking and the influence of the tobacco industry in the Commission. Furthermore, the widely publicised leaked documents alerted MEPs to the tobacco industry's tactics, and the possibility that any contact with industry might ultimately be made public.

Consistent with previous research,^19^ ^21^ ^22^ ^142^ we show that the EU's approach to IA and Smart Regulation favours corporate interests over public concerns and economic over health considerations,^142^ ^143^ and can be used to delay and ultimately prevent public health legislation. In contrast, FCTC Article 5.3, which aims to prevent industry influence on policymaking, is poorly understood and inadequately implemented. The Smart Regulation tools must be reviewed to ensure they serve the public and not just corporate interests, uphold Article 5.3, particularly in parts of the Commission not responsible for health and in the European Parliament, and fulfil the EU's broader commitment to transparent policymaking. Evidence that the tobacco industry relied on high-profile former EU officials to secure influence reveals a need to revisit rules on the employment of former Commission staff.^144^ ^145^ With a new Parliament and Commission recently appointed, including the addition of a new Commissioner for Smart Regulation clearly signalling a prioritisation of this agenda, these reviews are urgently needed.

**What this paper adds**

- This paper demonstrates that third party actors have become an increasingly important element of tobacco industry lobbying and play a central role in attempts to subvert European Union (EU) tobacco control policies.
- During the Tobacco Products Directive (TPD) review, tobacco industry access and influence was secured via the highest echelons of the European Commission, the Secretariat General, the Legal Service and Barroso's Cabinet.
- Intervention by these elements of the Commission led both to the removal of the two provisions from the TPD text that industry was most concerned about—plain packaging and a point of sales display ban—and to repeated delays to its progress through the Commission.
- These interventions followed repeated, undisclosed contact between senior Commission officials and the tobacco industry, signalling that Framework Convention on Tobacco Control (FCTC) Article 5.3 is poorly understood and implemented in the Commission, despite it being a signatory to the Treaty since 2005.
- This first assessment of how the Smart Regulation agenda affects EU tobacco control policymaking since the system was fully implemented confirms previous concerns that Smart Regulation enables corporate influence, and may thereby undermine EU public health policy.

## Supplementary Material

Web appendix1

Web appendix2
