# Supplementary material for: The revision of the 2014 European tobacco products directive: an analysis of the tobacco industry's attempts to ‘break the health silo’
Source: Tob Control. 2015 Feb 24;25(1):108–17. doi: 10.1136/tobaccocontrol-2014-051919 (PMC4669229; doi:10.1136/tobaccocontrol-2014-051919)
Supplement: Web appendix1 [file tobaccocontrol-2014-051919-s1.pdf]

Appendix 1 Freedom of Information requests that informed this study

Summary of Freedom of Information requests helped to inform the timeline, submitted by other parties and other members of the Tobacco Control Research Group (TCRG) as part of the ongoing monitoring of the lobbying activities of the tobacco industry

Date submitted	Relevant Directorate General/ Cabinet	Applicant	Number of docs released	Reference
21 Jun 11	Sec Gen	Smokefree Partnership	4	2011/3378
6 Jul 11	Bureau of European Policy Advisors	Fiona Godfrey	3	2011/3676
6 Jul 11	ENTR	Fiona Godfrey	19	2011/3677
6 Jul 11	Trade	Fiona Godfrey	6	2011/3678
27 Apr 12	SANCO	TCRG	47	2012/2163
19 Oct 12	Sec Gen	Corporate Observatory Europe	4	2012/4873
19 Oct 12	Barroso	TCRG	8	2012/4848
19 Oct 12	Barroso	Corporate Observatory Europe	18	2012/4874
2 Nov 12	Legal	TCRG	1	2012/5058
9 Nov 12	Sec Gen	TCRG	20	2012/5130
21 Jan 13	Sec Gen	Corporate Observatory Europe	1	2013/243
21 Jan 13	Sec Gen	Corporate Observatory Europe	4	2013/246
6 Feb 13	Legal	Corporate Observatory Europe	11	2013/0593
27 Mar 13	Sec Gen	Corporate Observatory Europe	6	2013/1860
27 Mar 13	Barroso	Corporate Observatory Europe	3	2013/1682
4 Feb 2014	SANCO	TCRG	1	2014/588

Summary of Freedom of Information requests submitted by authors relating to correspondence between the European Commission and the tobacco industry, private sector organisations and lobby groups relating to the Tobacco Products Directive (TPD)

Date submitted	Relevant Directorate General/ Cabinet	Requested information	Nr of docs released	Reference
23 Jul 13	Legal	Specifically contact with Michele Petite from Nov 2012	0	2013/3892
23 Jul 13	Sec Gen	Correspondence on TPD with third parties from 2010 to 2013	5	2013/3906
23 Jul 13	Trade	Correspondence on TPD with specific companies Including Swedish Match, CECCM, ESTOC between 2010 and 2013	49	2013/3874
25 Jul 13	Markt	Correspondence on TPD with BusinessEurope and VNO NCW since 2012	0	2013/3926
25 Jul 13	Legal	Correspondence on TPD with BusinessEurope and VNO NCW since 2012	0	2013/3911
25 Jul 13	Sec Gen	Details of six meetings with tobacco industry between 2010 and 2012	0	2013/3910
25 Jul 13	ENTR/ Tajani	Correspondence on TPD with BusinessEurope, VNO NCW, and DCI Group since 2012	0	2013/3927
25 Jul 13	Oettinger	Correspondence on TPD with BusinessEurope, VNO NCW, and	0	2013/3913

		DCI Group since 2012		
9 Aug 13	UK DH	PMI's submission to DH of its views on the TPD, and other correspondence b/w DH and PMI on TPD	5	In public domain
21 Aug 13	Barroso	Correspondence between Cabinet member Antonia Jose Cabral and tobacco industry, APED, CECCM, Bell Pottinger, DCI Group and Business Europe	0	2013/4322
21 Aug 13	RTD/Geoghan-Quinn	Correspondence on TPD with tobacco industry, specific national governments (SW, PL, RO, BL, CZ and GR) and Polish Chamber of Commerce	1	2013/4324
27 Nov 13	AGRI	Correspondence on TPD with private sector organisations and lobby groups from 2009	44	2013/5961
27 Nov 13	EMPL	Correspondence on TPD with private sector organisations and lobby groups from 2009	7	2013/5962
27 Nov 13	REGIO	Correspondence on TPD with private sector organisations and lobby groups from 2009	0	2013/5963
27 Nov 13	ENTR	Correspondence on TPD with private sector organisations and lobby groups from 2009	53	2013/5964
27 Nov 12	Markt/ Barnier	Correspondence on TPD with private sector organisations and lobby groups from 2009	23	2013/5959 2013/5969
27 Nov 13	Hahn	Correspondence on TPD with private sector organisations and lobby groups from 2009	28	2013/5970
27 Nov 13	Andor	Correspondence on TPD with private sector organisations and lobby groups from 2009	11	2013/5971
27 Nov 13	Ciolos	Correspondence on TPD with private sector organisations and lobby groups from 2009	39	2013/5973
27 Nov 13	Barroso	Correspondence on TPD with private sector organisations and lobby groups from 2009	24	2013/5976
27 Nov 13	Tajani	Correspondence on TPD with private sector organisations and lobby groups from 2009	19	2013/5972
27 Nov 13	Semeta	Correspondence on TPD with private sector organisations and lobby groups from 2009	0	2013/5975
27 Nov 13	Almunia	Correspondence on TPD with private sector organisations and lobby groups from 2009	22	2013/5977
27 Nov 13	Reding	Correspondence on TPD with private sector organisations and lobby groups from 2009	20	2013/5978
27 Nov 13	Lewandowski	Correspondence on TPD with private sector organisations and lobby groups from 2009	41	2013/5979
27 Nov 13	Rehn	Correspondence on TPD with private sector organisations and lobby groups from 2009	7	2013/5980
4 Dec 13	COMP	Correspondence on TPD with private sector organisations and lobby groups from 2009	19	2013/6098
4 Dec 13	JUST	Correspondence on TPD with private sector organisations and lobby groups from 2009	8	2013/6099
4 Dec 13	Eurostat	Correspondence on TPD or tobacco market with third parties from 2010	0	2014/642
4 Feb 14	OLAF	Correspondence on TPD with private sector organisations and lobby groups from 2010	0	2014/643
