# Supplementary material for: The revision of the 2014 European tobacco products directive: an analysis of the tobacco industry's attempts to ‘break the health silo’
Source: Tob Control. 2015 Feb 24;25(1):108–17. doi: 10.1136/tobaccocontrol-2014-051919 (PMC4669229; doi:10.1136/tobaccocontrol-2014-051919)
Supplement: Web appendix2 [file tobaccocontrol-2014-051919-s2.pdf]

Appendix 2 Associations and private companies involved in the campaign against stricter measures in the TPD

Associations and (non-TTC) companies that lobbied EU policy makers to oppose stronger TPD measures (either via correspondence, meetings, working groups, statements and petitions)	Geography	Sources
**BUSINESS AND TRADE ASSOCIATIONS**		
ACC (association of Communication Companies)	Belgium	1049
Acotex (Sociedad Cooperativa Ult Grado)	Spain	1049
AČRA (Asociace Českych reklamnich agentur a marketingové komunikace)	Czech Republic	1049
AFUBRA (Associação dos Fumicultores do Brasil)	Brazil	1049
ALEC (American Legislative Exchange Council)	US	1049
AMCHAM EU (American Chamber of Commerce)	US	PMI, 1049
AMCHAM Germany	Germany	1049
AMCHAM Italy	Italy	1049
ANCMMER (Asociaţia Naţională a Comercianţilor Mici şi Mijlocii din România)	Romania	1049
APRAM (Association des Praticiens du Droit des Marques et des Modèles)	International	Media, 1049
ASAJA (Asociación Agraria Jóvenes Agricultores)	Spain	1049
ASINCA (Asociacion Industrial de Canarias)	Spain	1049
Association des Commercants en Gros des Produits de Tabac du Piree	Greece	1049
Association of Independent Newsagents in Wallonia and Brussels	Belgium	1049
Association of tobacco kiosks and retailers of Athens	Greece	1049
BASCAP (Business Action to Stop Counterfeiting and Piracy)	International	Minutes, Parliamentary reports
BDI (Bundesverband der Deutschen Industrie)	Germany	1049*
BdZ (Bundesverband der Zigarrenindustrie)	Germany	Commission report, 1049
BLL (Bund der Lebensmittelrecht)	Germany	1049
BMM (Beneluxvereniging voor merken and modellenrecht)	Benelux	Media, 1049
BTWE (German National Federation of Tobacco Retailers)	Germany	Media, 1049
Burley Tobacco Growers Cooperative Association Lexington	US	1049
BusinessEurope	EU	PMI, Minutes, 1049, Parliamentary report
CCCI (Cyprus Chamber of Commerce and Industry)	Cyprus	1049
CCOO (Comisiones Obreras de Canarias)	Spain	1049
CEBRE (Czech Business Representation to the EU in Brussels)	Czech Republic	1049*
CECCM (Confederation of European Community Cigarette Manufacturers)	EU	Minutes, Commission & Parliamentary reports, 1049
CEOE (Confederación Española de Organizaciones Empresariales)	Spain	1049
CEDT (Confédération Européenne des Détaillants en Tabac)	EU	PMI, Media, 1049, Minutes, Parliamentary report, NGO report
CEPYME (Confederación Española de la Pequeña y Mediana Empresa)	Spain	1049
CLC (Commerce Transport Services)	Luxembourg	1049
Coalition for fine paper industry	EU	1049
Confédération des Buralistes de France	France	1049
Confederazione Nazionale Coldiretti	Italy	1049
Confindustria (Confederazione Generale dell'Industria Italiana)	Italy	1049
CGPME (Confederation Generale des Petites et Moyenne Entreprises)	France	1049
Copa (Cogeca)	EU	PMI, Minutes
CSNA (Convenient Stores and Newsagents Association)	Ireland	1049
DI (Confederation of Danish Industry)	Denmark	1049*
Dom Polski Wschodniej w Brukseli (East Poland House)	Poland	1049
DVAI (Deutscher Verband der Aromenindustrie e. V.	Germany	Minutes
EACA (European Association of Communications Agencies)	EU	1049, Parliamentary report

ECAT (Emergency Committee for American Trade)	US	1049
ECITA (Electronic Cigarette Industry Trade Association)	UK	Minutes, Parliamentary reports, NGO report
ECMA (European Cigar Manufacturers Associations	EU	Minutes, 1049, Commission report, Legacy
ECMA (European Carton Makers Association)	EU	Parliamentary reports, Minutes, 1049
ECTA (European Communities Trade Mark Association)	EU	PMI Parliamentary report, media, 1049
EFFA (European Flavour Association)	EU	Minutes, 1049
EFFAT (European Federation of Food, Agriculture and Tourism Trade Unions)	EU	PMI, Minutes, 1049
EMMA (European Magazine Media Association)	EU	Minutes, 1049
ERA (European Rotogravure Association)	EU	Minutes
ERPA (European Rolling Paper Association)	EU	Minutes
ESTA (European Smoking Tobacco Association)	EU	Minutes, 1049
ESTOC (European Smokeless Tobacco Council)	EU	PMI, Minutes, Commission & Parliamentary reports, 1049, media
ETRC (European Travel Retail Confederation)	EU	Minutes
ETV (European Tobacco Wholesalers Association)	EU	Parliamentary report, Minutes
FB (American Farm Bureau Federation)	US	1049
FEB-VBO (Federation of Enterprises in Belgium)	Belgium	1049*
Federation of Professional Renter Kiosks and Tobacconists of Greece	Greece	1049
FEDIL (Business Federation Luxembourg)	Luxembourg	1049
FEDMA (Federation of European Direct and Interactive Marketing)	EU	Parliamentary report
FETRATAB (European Federation of Tobacco Processors)	EU	PMI, Minutes, 1049
FIT (Federazione Italiana Tabaccai)	Italy	NGO report, 1049
FNCT (Federacion Nacional de Cultivadores de Tabaco)	Spain	1049
FRANCE TABAC (Union de Sociétés Coopératives Agricoles)	France	1049
FWD (Federation of Wholesale Distributors)	UK	Media
Forest (UK Smoker's Rights Group	UK	Minutes, Parliamentary report, blogs, website, media
GAMA (Global Acetate Manufacturers' Association)	US	PMI, Parliamentary reports, Minutes, 1049
GITES (Groupement des Industriels Européens du Tabac	EU	Minutes
GRUR (German Association for the Protection of Intellectual Property)	Germany	1049
IBEC (Irish Business and Employers confederation)	Ireland	1049*
ICC (International Chamber of Commerce)	International	Parliamentary report
ICC Lithuania	Lithuania	1049
IPA (Institute of Practitioners in Advertising)	UK	1049
IPH Krakowie (Izba Przemyslowo-Handlowa w Krakowie)	Poland	1049
ITGA (International Tobacco Growers Association)	International	Media, NGO report, 1049
IV (Vereinigung der österreichischen Industrie-Industriellenvereinigung)	Austria	1049
KH (Komitet Handlu Krajowej Izbie Gospodarczej)	Poland	1049
KIG (Krajowa Izba Gospodarcza)	Poland	1049
KPH (Kongregacja Przemystowo-Handlowa)	Poland	1049
KSPT (Krajowe Stowarzyszenie Przemysłu Tytoniowego)	Poland	Parliamentary report
LPK (Lithuanian Confederation of Industrialists)	Lithuania	1049*
MADOSZ (Magyar Dohánytermelök Országos Szövetsége)	Hungary	NGO report, media, 1049
MARQUES (The European Association of Trade Mark Owners)	EU	PMI, media, 1049
Medef (Mouvement des Enterprises de France)	France	1049
Mesa del Tabaco	Spain	1049
NAM (National Association of Manufacturers)	US	1049
NAT-2010 (National Tobacco Growers Association)	Bulgaria	Parliamentary report
National Brand Association	Unknown	1049
NBL (Naerbutikkernes Landsforening)	Denmark	1049
NDM (Näringslivets Delegation för Marknadsrätt)	Sweden	1049
NFRN (National Federation of Retail Newsagents)	UK	Media, 1049

NFTC (National Foreign Trade Council)	US	1049
NRZHIU (Naczelna Rada Zrzeszeń Handfu i Usług)	Poland	1049
NSO (Brancheorganisatie voor de tabaksdetailhandel)	Netherlands	1049
NSZZ Solidarność	Poland	1049
NTRG (Nordic Travel Retail Group)	Scandinavia	Minutes
OITAB (Organización Interprofesional del Tabaco de España)	Spain	1049
Organizatia Profesionala Pe Filiera de Tutun	Romania	1049
PIH (Polska Izba Handlu)	Poland	Parliamentary reports, Minutes, 1049
POHID (Polska Organizacja Handlu i Dystrybucji)	Poland	1049
Polish Confederation Lewiatan	Poland	Parliamentary report, 1049
Polish tobacco farmers association	Poland	Parliamentary report
Prodipresse (Association of Independent Newsagents in Wallonia and Brussels)	Belgium	1049
PZPT (Polski Zwiazek Plantatatorow Tytoniu)	Poland	1049
SEEHT (Greek Electronic Cigarette Trade Association)	Greece	Parliamentary report, Minutes
SEV (ΣΕΒ σύνδεσμος επιχειρήσεων και βιομηχανιών)	Greece	1049*
SNIAA (Syndicat National des Industries Aromatiques Alimentaires)	France	Parliamentary reports, Minutes
SSI (Stichting Sigarettenindustrie)	Netherlands	Legacy
Swedish Food Federation	Sweden	Minutes
Swedish National Association for Convenience Stores and Fast Food	Sweden	1049
Swedish Trade Federation (Svensk Handel)	Sweden	Minutes
Svenskt Naringsliv	Sweden	1049*
Tabacos de Cáceres cooperative	Spain	1049
TABD (Transatlantic Business Dialogue)	International	1049
TAMA (Tobacco Association of Malawi)	Malawi	1049
TGANC (Tobacco Growers Association of North Carolina)	US	1049
TVECA (Tobacco Vapor Electronic Cigarette Association)	EU	Minutes, Parliamentary report
UK Packaging Industry Group	UK	Media
Unión de Asociaciones de Estanqueros de Espana	Spain	1049
UNION-IP (Association of European Practitioners in Intellectual Property)	EU	Media
UNITAB (European Tobacco Growers Association)	EU	PMI, Parliamentary report, Minutes, 1049, NGO report, media
UNITAB Italia	Italy	1049
Unite	UK	Parliamentary reports
USCIB (US Council for International Business)	US	1049
VCPO (Austrian Retailers Association)	Austria	Media, 1049
VdR (Verband der deutschen Rauchtabakindustrie e. V)	Germany	1049
VDZ (Verband der Deutscher Zeitschriftenverleger)	Germany	Minutes, 1049
VEA (Vereniging van communicatieadviesbureaus)	Netherlands	1049
VFP (Vlaamse Federatie voor persverkopers)	Belgium	1049
VNK (Vereniging Nederlandse Kerftabakindustrie)	Netherlands	Legacy
VNO-NCW (Confederation of Netherlands Industry and Employers)	Netherlands	1049*, Legacy
VNS (Nederlandse Vereniging voor de Sigarenindustrie)	Netherlands	Legacy
WKO	Austria	1049
ZAW (Zentralverband der Deutschen Werbewirtschaft)	Germany	1049
ZO NOS PPP (Independent Trade Union of Workers in the Food Industry and Allied Trade)	Czech Republic	1049
ZZPPT (Federacja Zwiazkow Zawodowych Pracownikow Przemyslu Tytoniowego w Polsce)	Poland	1049
**COMPANIES**		
Addkeshaw Goddard LLP	UK	Parliamentary report
Amcor	Australia	1049
Arnold & Porter LLP	US	1049
Bell Pottinger	UK	1049
Blakemore Wholesale	UK	Media
Cadwalader, Wickersham & Taft LLP	US	1049
C&I (Communications & Institutions)	France	1049

Clifford Chance	France	Commission report, 1049
Cross Media PR	Poland	1049
DCI	International	Minutes
Delfortgroup	Austria	1049
Deltafina	Italy	1049
Deutsche Benkert GmbH & Co KG	Germany	Parliamentary report, 1049
Hogarth Chambers	UK	1049
Hume Brophy	International	Parliamentary reports
IME (Institute for Market Economics)	Bulgaria	1049
Interel European Affairs	Belgium	Parliamentary reports
Kreab Gavin Anderson	Belgium	Parliament report, 1049
Luther Pendragon Brussels	Belgium	NGO report
MANE	France	PMI, Parliament report
Mayr-Melnhof Group	Austria	1049
Media Intelligence Partners	UK	Parliament report
MMM (Medien Marketing Meinsen)	Germany	1049
MR Business Services	UK	Parliament report
Monopolverwaltung GmbH	Austria	1049
Pappas & Associates	Belgium	NGO report, 1049
Paynes Security	UK	Parliamentary report
SAT Tabacos de Talayuele	Spain	1049
SWM	US	1049
The European House Ambrosetti	Italy	1049
THN Ltd	UK	Parliament report
Weidenhammer Packaging Group	Germany	Minutes, Parliament reports
World Wide Tobacco España	Spain	1049
Work Services SA	Poland	1049

Note: Excluded from the analysis are health advocates, pharmaceutical associations/companies, and e-cigarette associations/companies with no apparent links with the tobacco industry.
